# Saliva Lipids May Determine Alprazolam Toxicity: An FTIR Study

**DOI:** 10.5812/ijpr-146675

**Published:** 2024-08-20

**Authors:** Arezou Mahdavinejad, Shahin Shadnia, Kiana Farhadinejad, Golrokh Farnam, Farshad H. Shirazi

**Affiliations:** 1 Toxicological Research Center, Excellence Center of Clinical Toxicology, Loghman Hakim Hospital, Shahid Beheshti University of Medical Sciences, Tehran, Iran; 2 Department of Toxicology and Pharmacology, School of Pharmacy, Shahid Beheshti University of Medical Sciences, Tehran, Iran; 3 Pharmaceutical Sciences Research Center, Shahid Beheshti University of Medical Sciences, Tehran, Iran

**Keywords:** Alprazolam, Toxicity, FTIR, Saliva, Lipids, Poison Control, Diagnostic Method

## Abstract

**Background:**

Alprazolam, a commonly prescribed benzodiazepine, poses risks of toxicity and severe withdrawal symptoms. There is an urgent need for a rapid and sensitive diagnostic method for detecting alprazolam poisoning.

**Objectives:**

This study aimed to detect alprazolam poisoning through Fourier-transform infrared (FTIR) analysis of saliva, addressing the need for a quick, cost-effective, and sensitive diagnostic method for poison control and differential diagnosis.

**Methods:**

Saliva samples were collected from 45 individuals with benzodiazepine toxicity, therapeutic consumption, and normal health status, as well as from a control group. The samples were analyzed using FTIR spectroscopy. The resulting spectra were processed with OriginPro software, and statistical analyses were performed using receiver operating characteristic (ROC) and analysis of variance (ANOVA).

**Results:**

The average age of the studied population was approximately 45 years, with women being the most affected by poisoning. Fourier-transform infrared analysis revealed significant differences in the structure of lipids between poisoned individuals, therapeutic receivers, and healthy individuals (P < 0.0001).

**Conclusions:**

Fourier-transform infrared analysis of saliva is a fast and accurate method for diagnosing alprazolam poisoning within minutes, enabling prompt and appropriate treatment during critical life-threatening situations. This non-invasive technique has the potential to guide treatment staff toward effective treatment options.

## 1. Background

Alprazolam is the most commonly prescribed benzodiazepine and psychotropic medication in the United States. In 2019, of the 92 million benzodiazepine prescriptions filled in pharmacies, 38% were for alprazolam (1). Despite concerns about its potential for misuse, it continues to be prescribed. Alprazolam is known to cause more severe withdrawal symptoms than other benzodiazepines, even when tapered according to manufacturer guidelines (2). According to national emergency department (ED) visit data, alprazolam is the second most common prescription medication and the most commonly involved benzodiazepine in ED visits related to drug misuse (3).

Extended use of benzodiazepines, including alprazolam, can pose risks beyond addiction. Alprazolam, in particular, is known for its heightened toxicity. The classic presentation in patients with isolated benzodiazepine overdose includes central nervous system (CNS) depression with normal or near-normal vital signs. Many patients will still be arousable and even able to provide a reliable history. Classic symptoms include slurred speech, ataxia, and altered mental status (4). Considering the negative effects of alprazolam, there is an urgent need to develop a simple yet highly sensitive technique for diagnosing alprazolam toxicity. 

The most reliable methods used in forensic laboratories to identify and quantify BZDs include gas chromatography (GC) coupled with an electron-capture detector or a mass spectrometer (MS), and HPLC. However, in rapid measurements and poisoning emergencies, immunoassay is the most common method (5). Immunoassay methods are not reliable for measuring alprazolam levels because this drug and its metabolites have low cross-reactivity with antibodies. Moreover, there is a risk of cross-reaction with other drugs that share a similar structure, such as antihistamines. The accuracy of each measurement kit also varies, and higher accuracy may lead to more false positive results (6, 7).

Alprazolam analysis can also be challenged by the interference of novel designer benzodiazepines, such as 4ʹ-chloro deschloroalprazolam, which is an isomer of alprazolam with similar physicochemical properties and mass spectra. This interference can lead to misidentification and inaccurate quantification of alprazolam in casework samples. Therefore, forensic laboratories need to develop specific methods to differentiate alprazolam from its analogs, such as using different chromatographic conditions, ion transitions, or reference standards (8).

Biomedical sample analysis widely uses Fourier-transform infrared (FTIR) spectroscopy (9). This technique relies on the absorption of infrared light through vibrational transitions in covalent bonds. The intensities of the absorption provide quantitative data, while the frequencies indicate the properties and structure of the bonds. Fourier-transform infrared spectroscopy detects the absorption modes in the mid-infrared region (400 - 4000 cm^-1^) through sharp bands. Biomolecules such as proteins, nucleic acids, lipids, and carbohydrates have distinct absorption patterns and spectral profiles, resulting in highly specific vibration bands in the mid-infrared region. This makes FTIR spectroscopy a simple, rapid, and noninvasive measuring technique that uses clear characteristic wavebands as spectral markers for a sample (10).

Saliva is a valuable biological fluid that can be used to assess the health status of the oral cavity and the entire body. Saliva contains various biomarkers that can indicate the presence or progression of various diseases and infections. One of the techniques that can be applied to saliva analysis is FTIR spectroscopy, which uses infrared radiation to measure the molecular vibrations of saliva samples. This technique is advantageous because it does not require invasive procedures or complex sample preparation, unlike other methods such as blood tests or polymerase chain reaction (PCR). However, one of the challenges of using saliva as a specimen for FTIR analysis is the low concentrations of salivary components, which can affect the quality and accuracy of the spectral data. Therefore, high-precision analytical methods are needed to overcome this limitation and obtain reliable results from saliva FTIR analysis (11).

## 2. Objectives

The aim of this study was to detect alprazolam poisoning by analyzing changes in a specific region of saliva using FTIR, presenting a quick, non-invasive, cost-effective, and sensitive method to diagnose alprazolam toxicity. This method could be useful in hospital emergency and toxicology departments for rapid diagnosis.

## 3. Methods

### 3.1. Sample Collection

The sample collection took place at Loqman Hakim Hospital in Tehran. During this experiment, 500 patients suspected of benzodiazepine poisoning and admitted to the emergency room of Loqman Hakim Hospital were examined. Based on the inclusion criteria, a total of 45 samples were included in this study.

Participants were divided into three groups in this study. One of the main exclusion criteria was any serious underlying pathological condition (such as poisoning with other drugs, active or chronic disease, or a positive PCR test for COVID-19). Blood tests were conducted to check the general health condition of the patients, and 45 samples were selected for further studies. Inclusion criteria included a positive result for alprazolam in hospital laboratory tests (Vitrotec, Iran) and the appearance of clinical evidence examined by a medical toxicologist.

The first group consisted of healthy individuals. To be included in the study, these individuals had to meet the following criteria: They had not had a viral or bacterial infection, such as a cold, in the past two weeks; they had no history of taking benzodiazepines in the past two weeks; and they had no underlying disease requiring medication for management. Additionally, individuals who had consumed alcohol in the past 24 hours or smoked in the week before the samples were taken were excluded from the study.

The second group consisted of people taking a therapeutic dose of alprazolam, prescribed over an extended duration at therapeutic levels. To select patients who take alprazolam in therapeutic doses, a psychiatrist's office in Tehran was visited, and individuals with a history of alprazolam use at the time of sampling were selected. This process was similar to the one followed for the previous samples. Sample collection was conducted by a trained technician in the specialist's office, and the same criteria were considered for inclusion in the study. This means that participants had been receiving alprazolam for at least 14 days under the supervision of a specialist doctor. Not only had the concentration of the drug in their plasma reached a stable and therapeutic level, but any side effects and their impact on saliva content could also be recognized and observed.

For poisoned individuals, the main criteria for entering the study were the absence of underlying diseases and the exclusive use of alprazolam, without other drugs or narcotics causing poisoning.

In all groups, the following instructions were followed before sampling: 45 minutes had to pass since their last meal, and they had to rinse their mouths well with water ten minutes before sampling. A trained nurse working in the emergency room was responsible for collecting the samples, and the sampling process was closely supervised. Efforts were made to rinse the patients' mouths with water prior to sampling and to allow at least 20 minutes for their saliva to return to its normal state. Subsequently, saliva samples were obtained. The collected samples were stored in a refrigerator at a temperature of 3 - 4°C until spectrometry. Additionally, blood and urine samples from poisoned individuals were taken within 30 minutes of their admission to the hospital and were sent to the hospital laboratory for blood factor analysis and benzodiazepine detection.

A similar process was used to examine and select the control group. Individuals who matched the age range of the poisoned group, had no benzodiazepines in their urine, and were in good health according to their blood factors were chosen. Additionally, in the group of healthy individuals, PCR tests were performed to check for a negative result for COVID-19, which was prevalent at the time of the study.

This project followed all ethical principles and received approval from the ethics committee of Shahid Beheshti University of Medical Sciences (IR.SBMU.RETECH.REC.1402.279).

### 3.2. FTIR Spectroscopy

The samples were analyzed using the RAYLEIGH FTIR Spectroscope, model WQS-510, with the MIRacle ATR Spectrometer accessory. One of the advantages of this method is that it allows for the analysis of samples without requiring any special preparation. Therefore, for spectrometry, the saliva samples previously stored in the refrigerator were exposed to the device's infrared radiation. Spectrometry was conducted within 48 hours after the sampling process. Another advantage of using this accessory is that a sample size of less than 50 µL is sufficient to obtain the infrared spectrum. There is no need to centrifuge or dry the saliva sample, as it is analyzed in the same state as it was received from the patient. To ensure consistent and uniform observation of spectra, the device was configured with a resolution of 4 cm^-1^ and a total of 50 sample scans were performed. These parameters were chosen to optimize the spectrometry process and ensure reliable data collection.

### 3.3. Data Analysis

To analyze the interferogram, Essential FTIR^®^ portable software was used to convert the graphical data into a numerical format that could be easily manipulated and stored in an Excel file. However, the spectrum obtained from the interferogram was not pure, as it contained the influence of water molecules present in the saliva samples. To remove this unwanted influence, a distilled water spectrum was taken under the same experimental conditions as the saliva samples and subtracted from the saliva spectra of each sample. This subtraction process assumed that the saliva samples had a normal water content of 99%, meaning that 99% of the saliva sample was composed of water molecules (12). After eliminating the water influence, the spectrum was ready for further analysis using OriginPro 2022 software. This software provided various tools for enhancing the quality and accuracy of the spectrum, such as baseline correction, second derivatization, peak finding, and deconvolution. Baseline correction adjusted the spectrum to have a zero baseline, representing the background noise. Second derivatization applied a mathematical operation to the spectrum to highlight the peaks and valleys, which are the points of maximum and minimum intensity. Peak finding identified and labeled the peaks and valleys in the spectrum, corresponding to the wavelengths of the light absorbed or emitted by the sample. Deconvolution separated the overlapping peaks and valleys into individual components, revealing the chemical composition and structure of the sample.

By using these tools, the spectrum was transformed into a more informative and reliable source of data. To evaluate the results and establish a connection between the observed peaks in the spectrum, alprazolam poisoning, and the individual's health, two statistical analysis methods were used. Receiver operating characteristic (ROC) analysis was employed to generate a curve to assess the accuracy and sensitivity of the test in distinguishing false positive and true positive results, using GraphPad Prism 9.5.1 software. Additionally, a repeated *t*-test statistical analysis was conducted on the data to determine if there are differences in the averages of groups or if random chance plays a role in these differences. Essentially, this test compares variations between groups and variations within groups.

Furthermore, a multiple *t*-test analysis can be carried out to explore how two independent variables impact a dependent variable. Through these calculations, we can ascertain whether the individual effects of each variable on the dependent variable are evident and whether the two independent variables interact with each other. This approach aids in gaining insight into the relationships among the variables under study.

## 4. Results

All demographic information and clinical evaluations of the patients were collected. In the studied population, most participants were in the age group of 24 - 35 years. Among both genders, women accounted for 73% of the population and were the most affected by poisoning. The length of hospitalization varied depending on the severity of the poisoning and the use of other medications, ranging from a few hours to five days (Tables 1 and 2). While fatalities resulting from benzodiazepine use are infrequent, they may arise from drug interactions or underlying medical conditions. Only one fatality was recorded within the study group, with the remaining patients being discharged after hospitalization periods ranging from one to five days, either with the attending physician's approval or upon giving their personal consent (13). The afflicted individuals were categorized into three cohorts based on the severity of benzodiazepine poisoning, as outlined in Table 1. 

**Table 1.** Classification of Patients According to the Severity of Alprazolam Poisoning

Severity of Poisoning	No. (%)
**Severe**	4 (26.7)
**Moderate **	1 (6.6)
**Mild **	10 (66.7)

**Table 2.** Demographic Information of Participants

Variables	Mean ± SD or No. (%)
**Demographic Categories**	
**Gender**	
Female	33 (73.3)
Male	12 (26.7)
**Age (y)**	
Under 18	1 (2.2)
18 - 24	6 (13.3)
25 - 34	16 (35.5)
35 - 44	15 (33.3)
45 - 54	7 (15.5)
**Poisoned Participants Information**	
**Drug history**	Negative
**Addiction history**	Negative
**Approximate consumed dose (mg)**	11.36 ± 6.54
**Consumption time (h)**	4.86 ± 4.79
**Underlying disease **	-
**Number of days of hospitalization (day)**	1.29 ± 0.59

The patients were categorized based on the severity of drug poisoning: Individuals experiencing severe poisoning were labeled with code 1, those with moderate poisoning were marked with code 2, and patients with mild poisoning were designated with code 3. The majority of cases presented with mild poisoning. Benzodiazepine poisoning poses a significant risk of inducing rapid-onset metabolic disturbances. An observed consequence of benzodiazepine toxicity is somnolence, which has a higher propensity in elderly individuals and with elevated dosages, potentially progressing to varying degrees of coma. None of the subjects had any underlying disease. Notably, the dosage range associated with symptoms of poisoning in hospitalized individuals was estimated to be between 4 and 40 milligrams. The effective daily dose of alprazolam for treating different mental illnesses is 0.75 to 9 mg. It should be observed that higher doses may increase the chances of experiencing side effects. Typically, therapeutic levels of alprazolam in blood plasma lie between 0.005 and 0.05 mg/L, while the toxic plasma level is within 0.1 and 0.4 mg/L. An overdose has been reported with plasma concentrations ranging from 0.12 to 0.39 mg/L (14). A more comprehensive description of the patients' demographic information is provided in Table 2. 

**Figure 1.**
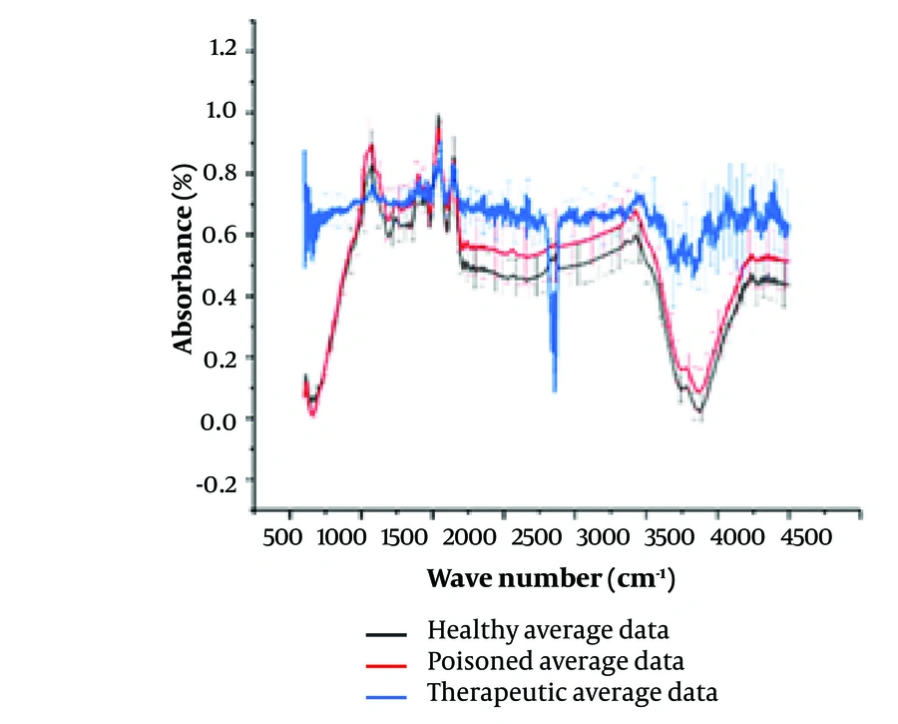
Comparison of the Fourier-transform infrared (FTIR) spectra from healthy, chronic therapeutic consumption, and poisoned persons’ saliva samples. Spectra in black represent the average of spectra taken from control group, while the red one shows the average spectra of poisoned group and the blue one from the patients taking Alprazolam as therapeutic medication. The spectra represent each group averages with standard deviations being as vertical lines.

Figure 1 presents the FTIR spectra of saliva samples from three distinct groups of individuals. It highlights the contrast between the average spectrum observed in healthy samples, those from individuals poisoned with alprazolam, and those who have consumed alprazolam in therapeutic doses. The graphs in Figure 1 depict the raw spectrum before baseline correction and region selection.

**Figure 2.**
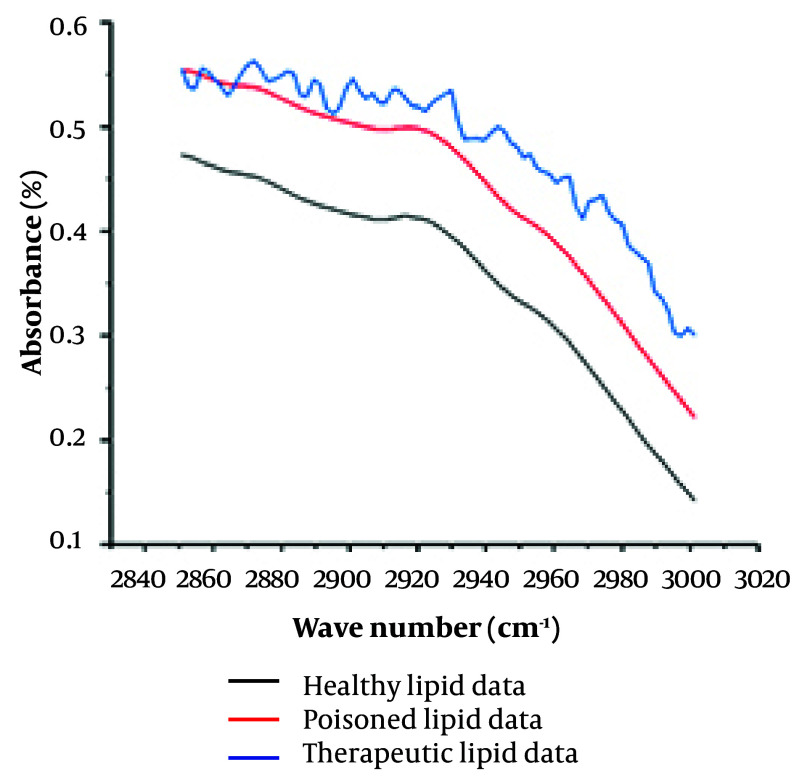
Spectrum outline processed and averaged from the three groups is depicted as follows: Blue signifies healthy individuals, red represents poisoned individuals, and black indicates those who were administered therapeutic doses.

Figure 2 illustrates the analysis timeline. It showcases the process of extracting hidden peaks, data processing, water spectrum removal, and baseline averaging across the three groups. Notably, the graph representing healthy individuals and those affected by poisoning exhibits a similar pattern. In contrast, the graph for individuals using alprazolam at therapeutic levels displays distinct peaks within this range.

**Figure 3.**
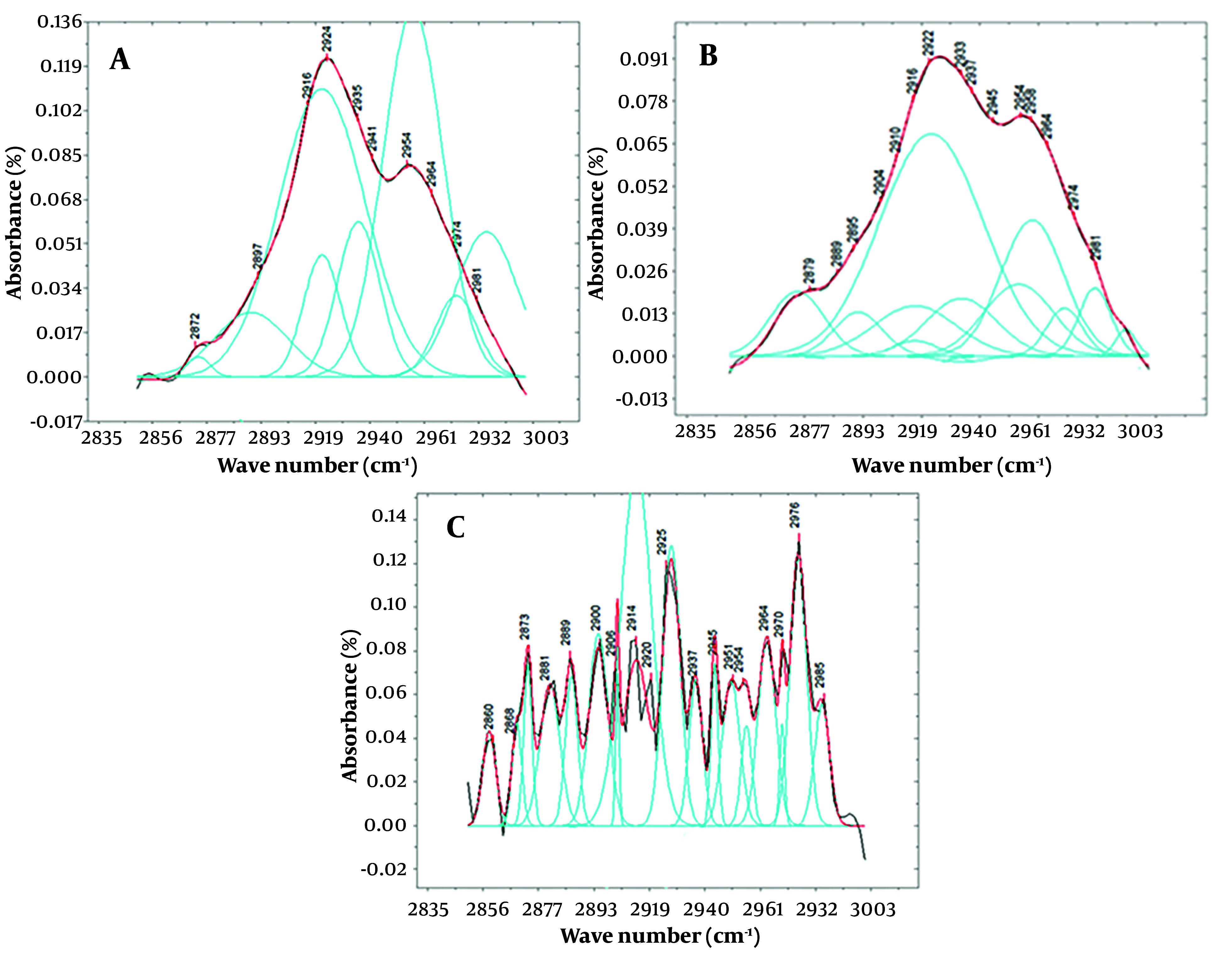
A, presents the deconvolution of the function derived from the saliva of a healthy volunteer after subtracting the baseline; while B, illustrates the deconvolution and peaks obtained from fitting the function derived from the saliva of poisoned patients, both within the range of 2850 to 3000 cm^-1^. Spectra of C is representing the same as “a” and “b” for the patients using alprazolam as a therapeutic agent. It's important to note that the y-axis in all of these graphs represents the intensity of the peaks, with the graph for the poisoned volunteers showing significantly lower intensity compared to the healthy volunteers. The color scheme in these graphs are as follows: The black line represents the original graph from which the baseline is subtracted, the red line depicts the fitted function, and the blue lines represent Gaussian functions, the sum of which forms the spectrum within this range.

Figure 3 displays deconvoluted peaks extracted from spectra between 2850 and 3000 wave numbers for the average saliva of each study group. While Figure 2 clearly shows differences in peak intensity, the specific areas where the peaks appear in individuals affected by poisoning and the control group can contribute to a more precise and accurate diagnosis.

In this context, Figure 3 represents the selected 2850 - 3000 cm^-1^ region after proper processing to better identify underlying peaks in the control, therapeutic consuming group, and poisoned group spectra. Notably, the spectral features related to lipids in the poisoned group exhibit irregularities due to the appearance of several discriminated peaks compared to the healthy group. Noteworthy peaks include a wavelength of 2901 cm^-1^ in healthy individuals, which splits into two peaks at 2900 and 2909 cm^-1^ in the poisoned group. Additionally, the saliva of poisoned subjects shows peaks at 2865, 2871, 2879, and 2992 cm^-1^, which are absent in healthy individuals. These spectral differences can aid in precise and accurate diagnostic assessments.

**Figure 4.**
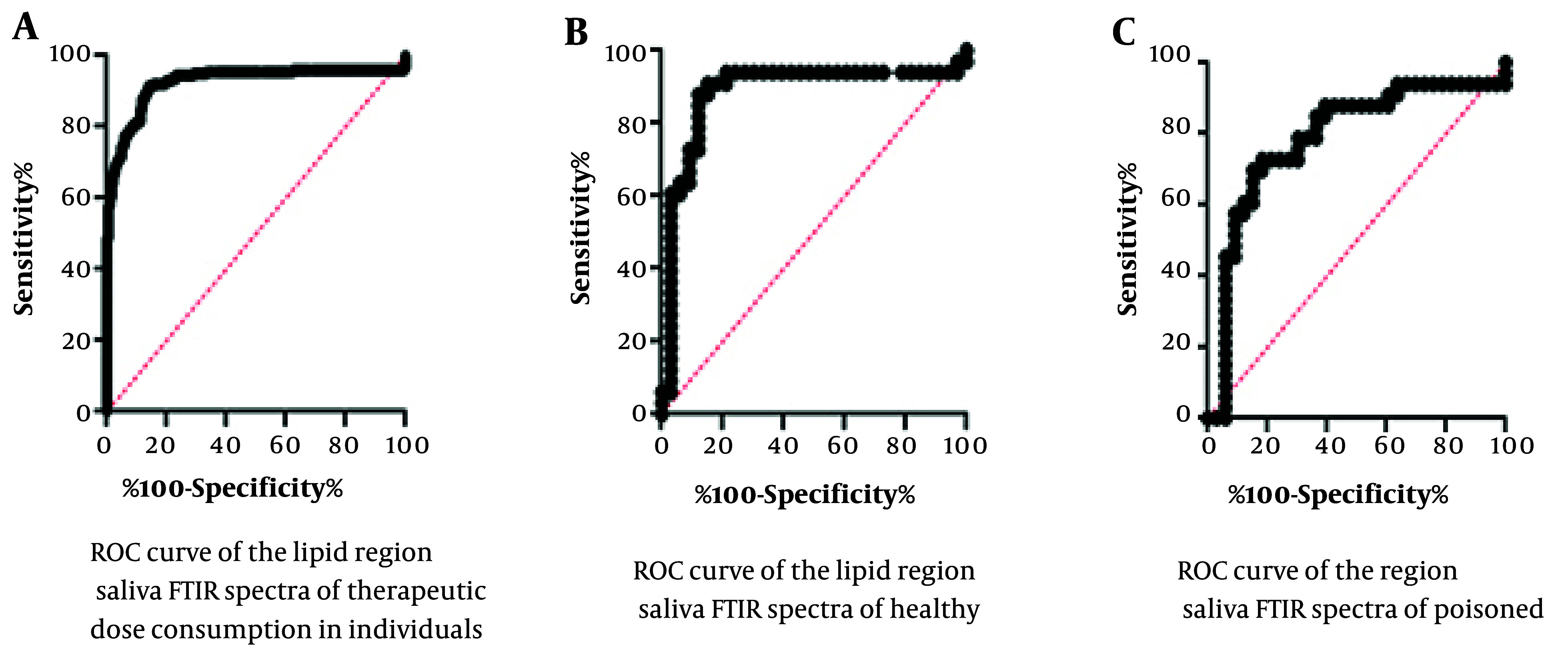
In this figure, the rock charts are utilized to compare the outcomes between different groups: A, those receiving a therapeutic dose of alprazolam; B, healthy individual; and C, poisoned individual. The x-axis of the graph in this evaluation denotes test sensitivity, while the y-axis represents test specificity.

The ROC statistical analysis results are presented in Figure 4 and Table 3. Figure 4 shows the specificity versus sensitivity of the assay by examining the cut-off values obtained from the test results, which indicate the reliability of the assay in distinguishing test numbers from control numbers. The area under the curve is 89.25%, with a calculated standard deviation of 1.79%. This indicates a high level of significance, highlighting the importance of the difference between positive and negative test results.

**Table 3.** The Statistical Data Obtained from the Rock Test for the Saliva Samples of Healthy, Therapeutic-Dose Consumers and Alprazolam-Poisoned Individuals’ Fourier-Transform Infrared Spectra

Variables	Area	SE	95% Confidence Interval	P-Value
**Healthy volunteers compared to poisoned volunteers**	0.8925	0.01791	0.8574 - 0.9276	< 0.0001 ^a^
**Healthy volunteers compared to therapeutic-dose consumer volunteers**	0.8843	0.04919	0.7879 - 0.9807	< 0.0001
**Poisoned volunteers compared to therapeutic-dose consumer volunteers**	0.7879	0.06022	0.6698 - 0.9059	< 0.0001

^a^ P-value < 0.05 indicates that the observed difference between groups is statistically significant.

The statistical analysis results of multiple *t*-tests for the intensity of peaks within the range of 2910 to 2920 cm^-1^ indicate statistically significant differences between the three groups: Non-use (healthy individuals), acute use (poisoned individuals), and chronic use (those administered alprazolam at therapeutic doses) (Table 4). 

**Table 4.** Significant Differences Between Deconvoluted Peaks of Different Groups at 2910 - 2920 cm^-1^

Compared Groups	P-Value ^a^
**Healthy vs. therapeutic**	< 0.000001
**Poisoned vs. therapeutic**	< 0.00001
**Healthy vs. poisoned**	< 0.0001

^a^ P-value < 0.05 indicates that the observed difference between groups is statistically significant (n = 15).

Through pairwise comparisons among the groups, significant differences were observed in the mean values of the paired comparisons. Specifically, when comparing the healthy and poisoned groups, distinct patterns of differences in mean values were identified between the two groups.

## 5. Discussion

The development of a robust method for the precise and swift diagnosis of poisonings is of paramount importance, particularly concerning drugs with pronounced side effects, toxicities, and a high incidence of poisoning, substance abuse, and addiction. During this critical window, timely interventions such as gastric lavage, dialysis, and antidote administration can significantly improve the patient’s condition. Swift initiation of treatment not only reduces the risk of complications but also expedites the patient’s recovery. However, existing laboratory and confirmatory techniques often entail prolonged processing times, require sophisticated instrumentation, and demand specialized personnel. Consequently, there is a pressing need for an automated and efficient diagnostic approach that can yield rapid and accurate results within minutes. This urgency extends to scenarios where patients are unconscious or lack immediate companionship. Developing an effective diagnostic technique capable of swift identification and differentiation of poisoning cases holds immense clinical significance, contributing to improved patient outcomes and streamlined healthcare delivery.

Fourier-transform infrared is a fast and accurate technique for analyzing various chemical and biological samples, which can be highly beneficial in clinical settings. Saliva analysis is a non-invasive, rapid, and accurate method that can aid in diagnosing illnesses or poisonings and guide treatment staff towards appropriate treatment options. Saliva is one of the most promising biological samples, with over 800 metabolites identified, and it is similar to serum in terms of complexity. The concentration of many diagnostic analytes in saliva correlates with their blood concentration (15). Saliva analysis can be used for the early detection and diagnosis of drug toxicities. In 2021, the biochemical composition and properties of saliva were analyzed by FTIR, investigating 34 indicators related to carbohydrates, proteins, minerals, and lipids metabolism. This expands the possibilities for interpreting FTIR spectra of saliva in biomedical applications (16). 

Many studies have been conducted on the simultaneous use of FTIR spectroscopy and saliva content measurements, yielding promising results in diagnosing diseases such as cancer, diabetes mellitus, chronic kidney disease (CKD), neonatal sepsis, and infectious diseases like COVID-19 (17-21). Other studies have investigated the application of saliva IR-spectroscopy in monitoring drug levels. These studies are significant in two ways: For real-time drug monitoring and personalized medicine, and for diagnosing drug poisoning (22). 

We used an FTIR instrument equipped with an ATR device. The advantage of using ATR for the spectroscopy of biological samples is its ability to better present the molecules' spectra in a water base, which might otherwise mask many spectral features of the sample due to the wide peak of water molecules. ATR use enables stronger absorbance by the sample due to the multiple points where the radiation interacts.

The quest for accurate and efficient methods to detect and quantify alprazolam, a widely used anxiolytic drug, has driven extensive research efforts. Commonly used methods include nuclear magnetic resonance (NMR), chromatography (GC-MS, HPLC, and TLC), immunoassay (ELISA, RIA, LFA, CEDEA, FPIA, and KIMS), and electroanalytical methods (voltammetry and potentiometry) (23). Our method focuses on the effects of alprazolam on saliva, rather than the drug itself. This approach is innovative and potentially faster and simpler, especially in emergency and poisoning centers.

HPLC is a commonly used method for the detection and quantification of benzodiazepines, including alprazolam. However, compared to our method, HPLC has a higher limit of detection (lower sensitivity) and requires more time to complete the detection process (24). GC-MS is another method used for alprazolam detection. While it can provide accurate results, it may not be as fast or simple as our FTIR method.

The distinctive advantage of FTIR lies in its ability to provide a concise and informative representation of peak presence or absence. Unlike other techniques that often necessitate specialized personnel for handling and result interpretation, FTIR offers a straightforward approach. By analyzing specific peaks within saliva samples, clinicians can swiftly arrive at preliminary judgments, facilitating timely interventions even in cases where patients are unconscious or lack immediate companionship.

What does FTIR detect in the patient's saliva? Is the result obtained due to the presence of alprazolam in the saliva, or due to the changes that occur in people's saliva after taking this drug? Although the chemical structure of alprazolam does not favor peaks in the selected lipid region of 2850 - 3000 cm^-1^, more clues might come from previous Raman spectroscopy studies of saliva from patients who used alprazolam. The result of a study conducted by Allqvist et al. in 2005 was that the traceable amounts of alprazolam in the saliva of a person who consumes this drug are at the trace level, with nanogram concentrations per milliliter of the person's oral fluids, making it very difficult to detect clear peaks in the rather complex composition of saliva (25). Therefore, it is likely that alprazolam poisoning affects the spectrum of saliva by changing its structure and composition rather than through the presence or secretion of detectable residue in the saliva.

Research has indicated that prolonged use of alprazolam in therapeutic doses can result in alterations in lipid and carbohydrate profiles, as well as changes in the composition of saliva. Studies suggest that the secretion of proline-rich proteins decreases with alprazolam use, potentially impacting the consistency of saliva by reducing the presence of compounds like mucin. This effect is not directly related to the pharmacological mechanism of alprazolam but could be considered a side effect of the drug. Nonetheless, changes in saliva composition might serve as an additional factor for more precise diagnostic considerations (26).

To ascertain the efficacy of our spectrometry method and subsequent spectral calculations in discerning between healthy individuals, those poisoned, and those receiving therapeutic doses, further investigations involving a larger population are imperative. Rigorous studies can provide robust evidence regarding the discriminatory power of this approach. Before applying necessary peak adjustments for analysis, preliminary observations were made concerning the lipid region of saliva spectra, specifically within the wave number range of 2850 to 3000 cm^-1^. Notably, all three groups—healthy individuals, those utilizing alprazolam at therapeutic levels, and the poisoned group—exhibited a consistent decreasing trend in this spectral domain. However, a more pronounced negative slope was observed in the poisoned and therapeutically dosed alprazolam groups. Intriguingly, the shape of the spectral graph differed markedly: While the poisoned group displayed a smooth profile, the other groups exhibited jagged patterns. Subsequent baseline definition across all three groups in this specific spectral region led to a convergence of the spectra, albeit with discernible variations. These findings underscore the potential of spectrometry as a rapid and informative diagnostic tool, even in scenarios where patients lack consciousness or immediate companionship (27, 28).

Before selecting the peaks using the OriginPro software, there may not have been much difference in the appearance of the fitted peaks between poisoned and healthy individuals, but the difference in the intensity of the peaks was easily recognizable. The peaks of poisoned individuals were observed to have much lower intensity than those of healthy individuals in this range. In individuals receiving a therapeutic dose, the peaks were less intense than in healthy individuals but appeared stronger than the peaks in the spectrum of poisoned individuals. 

Based on the observed changes in the saliva spectrum in the lipid region due to the consumption of this drug, and considering that the detected amounts of the drug in saliva are in the range of nanograms and can be disregarded, it can be inferred that one of the effects of this drug is related to the lipid profile in saliva. It appears that this drug somehow alters the lipid profile in saliva or the fats present in saliva (29, 30). For instance, the reduction in peak intensity in this area may be attributed to chemical reactions resulting in a decrease in the amount of fat in saliva. 

Analysis of variance (ANOVA) has confirmed that the peaks resulting from the lipid content of saliva from each of the poisoned, therapeutic consuming, and healthy groups are significantly different from each other, providing a good chance to diagnose toxicity from other groups. Receiver operating characteristic calculations and graphs have confirmed the validity and applicability of this technique for clinical and emergency applications as a fast, easy, and precise method.

### 5.1. Conclusions

Although this research is based on preliminary data with limited samples, it has shown that FTIR analysis of saliva might provide clues about the consumption of alprazolam in individuals at therapeutic or toxic levels. Notably, the technique does not measure alprazolam directly but analyzes saliva affected by alprazolam, which can discriminate between the three groups: Those who have not used alprazolam, those who have used it at therapeutic levels, and those who have used it at toxic levels. One limitation of this study is the potential interfering factors that might be discovered in future larger studies. 

The technique is simple, requires no sample preparation, and can provide results in a minute. Changes in the FTIR spectra of saliva in the region associated with lipids have confirmed its potential use for diagnostic purposes, supported by statistical calculations with sufficient accuracy and precision. This method could be particularly useful in emergency and poisoning centers. 

In comparison with other current techniques used for alprazolam detection in the body, such as HPLC, GC, mass spectrometry, and NMR, which are based on the detection and measurement of alprazolam in biological fluids, the advantage of this technique is its ability to categorize individuals as normal, therapeutic users, or poisoned based on the biological effects of alprazolam. This is the first time FTIR has been used in this manner.

## Data Availability

The dataset presented in the study is available on request from the corresponding author during submission or after publication.
